# Social Isolation, Health, and Health Care: Perspectives Among Older Adults Residing in Public Housing

**DOI:** 10.1093/geroni/igaa057.379

**Published:** 2020-12-16

**Authors:** Ruth Sanchez, Brooke Wagen, Whitney Williams, Elizabeth Jacobs

**Affiliations:** University of Texas at Austin Dell Medical School, Austin, Texas, United States

## Abstract

By 2035, U.S. adults > 65 will outnumber children. The growing lack of affordable housing combined with fixed incomes will lead to more older adults residing in public housing. Public housing authorities, in turn, will face growing health and social needs among their residents. In partnership with a local housing authority, we conducted a qualitative study to better understand the health and social needs of older adult public housing residents. We conducted semi-structured qualitative interviews with 27 older adults at two public housing sites in Austin, Texas; we asked about their experience of aging in public housing, their health, healthcare, and community life. Interviews were audio-recorded and transcribed; interviews were systematically coded and verified by a second coder. Themes were identified using comparative analysis. We interviewed 16 females and 11 males (mean age = 71.7 years). We identified three themes. Residents characterized good healthcare as that which is provided by physicians who are consistent educators that listen to residents’ primary concerns. They defined health as being mobile and lacking pain. Finally, they desire more, recurring opportunities to learn about health and connect interpersonally within their housing community; they perceive limited meaningful relationships as a significant contributor to poor health among residents. The older adult public housing residents in our study outlined what good health and healthcare looks like. These themes can be utilized to improve relationships between residents and their healthcare providers. Social isolation can be mitigated through public housing programming that promotes physical and mental acuity.

